# The Gamma Gap and All-Cause Mortality

**DOI:** 10.1371/journal.pone.0143494

**Published:** 2015-12-02

**Authors:** Stephen P. Juraschek, Alison R. Moliterno, William Checkley, Edgar R. Miller

**Affiliations:** 1 Department of Epidemioloy, Johns Hopkins Bloomberg School of Public Health, Baltimore, MD, United States of America; 2 The Welch Center for Prevention, Epidemiology and Clinical Research, Johns Hopkins University, Baltimore, MD, United States of America; 3 Division of General Internal Medicine, Department of Medicine, Johns Hopkins Medical Institutions, Baltimore, MD, United States of America; 4 Division of Hematology, Department of Medicine, School of Medicine, Johns Hopkins University, Baltimore, MD, United States of America; 5 Division of Pulmonary and Critical Care Medicine, Department of Medicine, School of Medicine, Johns Hopkins University, Baltimore, MD, United States of America; 6 Department of Biostatistics, Johns Hopkins Bloomberg, School of Public Health, Baltimore MD, United States of America; Azienda Ospedaliero-Universitaria Careggi, ITALY

## Abstract

**Background:**

The difference between total serum protein and albumin, i.e. the gamma gap, is a frequently used clinical screening measure for both latent infection and malignancy. However, there are no studies defining a positive gamma gap. Further, whether it is an independent risk factor of mortality is unknown.

**Methods and Findings:**

This study examined the association between gamma gap, all-cause mortality, and specific causes of death (cardiovascular, cancer, pulmonary, or other) in 12,260 participants of the National Health and Nutrition Examination Survey (NHANES) from 1999–2004. Participants had a comprehensive metabolic panel measured, which was linked with vital status data from the National Death Index. Cause of death was based on ICD10 codes from death certificates. Analyses were performed with Cox proportional hazards models adjusted for mortality risk factors. The mean (SE) age was 46 (0.3) years and the mean gamma gap was 3.0 (0.01) g/dl. The population was 52% women and 10% black. During a median follow-up period of 4.8 years (IQR: 3.3 to 6.2 years), there were 723 deaths. The unadjusted 5-year cumulative incidences across quartiles of the gamma gap (1.7–2.7, 2.8–3.0, 3.1–3.2, and 3.3–7.9 g/dl) were 5.7%, 4.2%, 5.5%, and 7.8%. After adjustment for risk factors, participants with a gamma gap of ≥3.1 g/dl had a 30% higher risk of death compared to participants with a gamma gap <3.1 g/dl (HR: 1.30; 95%CI: 1.08, 1.55; *P* = 0.006). Gamma gap (per 1.0 g/dl) was most strongly associated with death from pulmonary causes (HR 2.22; 95%CI: 1.19, 4.17; *P* = 0.01).

**Conclusions:**

The gamma gap is an independent risk factor for all-cause mortality at values as low as 3.1 g/dl (in contrast to the traditional definition of 4.0 g/dl), and is strongly associated with death from pulmonary causes. Future studies should examine the biologic pathways underlying these associations.

## Background

The “gamma gap” or globulins, i.e. the difference between total serum proteins and albumin measured from a comprehensive metabolic panel, is a frequently used clinical screening tool to assess for latent infection, malignancy, or autoimmune inflammatory diseases [1–4]. This is based on the observation that albumin accounts for the majority of total serum protein, while with viral infections, plasma cell malignancies, or autoimmune conditions there is an excess of immunoglobulins, raising the total amount of serum protein independent of albumin [4]. In fact, one study demonstrated that a higher gamma gap was a strong predictor for a positive serum or urine protein electrophoresis [1]. However, there is little evidence guiding application of the gamma gap in clinical practice. For example, an arbitrary value of 4.0 g/dl is considered a positive gamma gap even though there are no prospective studies examining gamma gap in association with clinical outcomes [5]. It is equally unknown whether the gamma gap is a risk factor of mortality independent of its commonly associated disease states (infection, malignancy, or inflammation).

The purpose of this study was: (1) to determine the level at which gamma gap is associated with an increased risk of mortality in a general US population; (2) to assess whether the gamma gap is associated with mortality independent of other common risk factors; and (3) to examine specific causes of death associated with the gamma gap. We hypothesized that the gamma gap would be associated with all-cause mortality at levels close to the traditional value of 4.0 g/dl. Further, we expected that this association would be independent of traditional risk factors and would be stronger with death from cancer.

## Methods

### Study Population

The NHANES surveys are large, cross-sectional studies conducted by the National Center for Health Statistics (NCHS). These surveys utilize a complex, multistage sampling design to represent the demographic constitution of the US adult population. We specifically used the interviews, physical examinations, and laboratory measurements of participants, age 20 or older, who visited the Mobile Examination Centers of the continuous NHANES 1999–2004. Participants <20 years of age (N = 15,189), lacking a comprehensive metabolic panel (N = 9,795), lacking covariates of interest (N = 1,068), or no follow-up time (N = 7) were excluded (note some participants were excluded for more than one of the aforementioned reasons). The NCHS Research Ethics Review Board approved the protocols for the conduct and execution of the NHANES and obtained written informed consent via consent forms [6].

### Gamma gap

A serum comprehensive metabolic panel was determined in all participants of NHANES 1999–2004 as part of the original protocol [6]. Analyses were performed with a Hitachi Model 704 multichannel analyzer (Boehringer Mannheim Diagnostics, Indianapolis, IN). Total protein was assessed with a colorimetric assay, while albumin was determined via a Bromocresol purple reagent, which binds selectively with albumin. The gamma gap was defined as the difference between total protein and albumin (Total Protein—Albumin). In this study, gamma gap was characterized as a continuous variable, as a dichotomous variable using multiple cut points between 2.5 g/dl (10^th^ percentile) and 4.3 g/dl (99^th^ percentile), and as a categorical variable based on quartiles.

### All-cause mortality and cause-specific mortality

The primary outcome from this study was mortality from any cause. Mortality status, time-to-event data (through December 31^st^, 2006), and underlying cause of death was ascertained using the NHANES, public-use linked mortality data. Death certificates from the National Death Index (including date of death and cause of death) were linked with NHANES study participants based on a probabilistic matching algorithm [6]. Cause of death was based on the International Classification of Diseases, Tenth Revision (ICD-10) guidelines [6]. Specific causes of death were: cardiovascular disease (ICD-10 code: I00-I078), cancer (C00-C97, D00-D48), pulmonary (J00-J98), and other (all deaths not from cardiovascular disease, cancer, or pulmonary disease).

### Risk factors related to mortality and the gamma gap

Model covariates were selected based on known clinical associations with mortality, low albumin states (liver disease or kidney disease), or inflammation. Age, gender, and race/ethnicity of all participants were obtained via self-report. Race/ethnicity categories were non-Hispanic white, non-Hispanic black, Mexican American, Hispanic, and Other. Hypertension was defined by a systolic blood pressure ≥140 mmHg or diastolic blood pressure ≥90 mmHg or use of antihypertensive medications [7]. A history of cancer was based on self-report of a health professional diagnosis. Body mass index (BMI) was calculated using weight and standing height measurements. Smoking status (never, former, or current) was based on self-report. Serum creatinine measures were standardized [8] and then used to estimated glomerular filtration rate (eGFR) with the Chronic Kidney Disease Epidemiology Collaboration equation [9]. Albuminuria was quantified using the albumin–creatinine ratio, expressed in mg/g. Total cholesterol (mg/dl), high-density lipoprotein (HDL) cholesterol (mg/dl), aspartate aminotransferase (AST, U/L), alanine aminotransferase (ALT, U/L), alkaline phosphatase (U/L), total bilirubin (mg/dL), C-reactive protein (mg/dl), white blood cell count (SI), and albumin (g/dl) were measured in serum specimens. The presence of hepatitis B virus core antibody and hepatitis C virus antibody were assessed in all participants and recorded as either positive or negative. Testing for human immunodeficiency virus (HIV) antibody was performed in participants age 20–49 per the NHANES protocol, using the Synthetic Peptide Enzyme Immunoassay for HIV-1 and HIV-2 [6].

### Statistical analyses

All analyses were performed in concordance with the NHANES complex sampling design, employing the sample weights, primary sampling units, and strata that accompanied each survey via the Taylor series (linearization) method. Baseline characteristics were expressed as means (SE) and proportions overall and across quartiles of the gamma gap. Causes of death were also tabulated by quartile of gamma gap. The distribution of the gamma gap was also examined across age and sex.

Weighted crude risk estimates were performed by determining the 5-year cumulative mortality rate across quartiles of the gamma gap. These were also assessed via Kaplan-Meier curves with trends across quartiles assessed via a logrank test. The distribution of the gamma gap by vital status was compared using kernel density plots and its median value was compared via an unweighted, two-sample, Kolmogorov–Smirnov equality-of-distributions test.

To evaluate different definitions of the gamma gap, we used multiple cut points between 2.5 and 4.3 g/dl (range chosen to represent percentiles 10–99), modeling it as a dichotomous variable. Weighted Cox proportional hazard models were used to compare the relationship between gamma gap with death from any cause. Models were nested in the following fashion. Model 1 was adjusted for age, sex, and race/ethnicity. Model 2 was adjusted for the covariates in Model as well as hypertension status, self-reported cancer, body mass index, smoking status, estimated glomerular filtration rate, albuminuria-to-creatinine ratio, total cholesterol, HDL-cholesterol, aspartate aminotransferase, alanine aminotransferase, alkaline phosphatase, total bilirubin, C-reactive protein, white blood cell count, hepatitis B core antibody status, hepatitis C virus antibody status, and serum albumin.

When evaluating cause specific mortality, gamma gap was modeled as a continuous variable, as a dichotomous variable (using the median, 95^th^ percentile, the traditional cutpoint of 4 g/dl, and the 99^th^ percentile) or categories based on quartiles. In these analyses, the fully adjusted, weighted Cox proportion hazards model (Model 2 above) was used to examine the association between gamma gap and death from any cause, death from cardiovascular disease, death from cancer, death from pulmonary disease, and death from all other causes (not cardiovascular, not cancer, not pulmonary). The continuous relationships between gamma gap and each of the causes of death were also evaluated with restricted cubic spline models centered at the median value, using Harrell’s recommended percentiles to determine three knot locations [10].

When modeled as quartiles, it was noted that the second quartile had a lower risk of mortality than the first quartile. As a result, we performed a sensitivity analysis using the second quartile as the reference (rather than the first quartile). We also performed sensitivity analyses in subpopulations restricted to participants without antibodies to HIV, hepatitis B, or hepatitis C at baseline.

## Results

The mean (SE) age was 46 (0.3) years; 51.6% were women, 72.8% were non-Hispanic white (Table 1), and the mean gamma gap was 2.99 g/dl (median, IQR: 3.0, 2.7 to 3.2). Age, the percentage of women, and all race/ethnicity groups except non-Hispanic whites were higher across quartiles of the gamma gap. Furthermore, hypertension, BMI, the percentage of never smokers, eGFR, albuminuria, AST, ALT, alkaline phosphatase, c-reactive protein, white blood cell count, and the percentage with HBV, HCV, or HIV antibodies were higher across quartiles of the gamma gap. In contrast, the percentage with self-reported cancer trended downward across quartiles of the gamma gap. Further, the percentages of both former and current smokers were lower across quartiles of the gamma gap. Mean total bilirubin concentrations were also lower across quartiles of the gamma gap. Neither total cholesterol nor HDL cholesterol levels differed across quartiles of the gamma gap. Details related to the distribution of gamma gap in the US population by age and sex may be found in **Table A in** S1 File. Baseline gamma gap levels did not vary substantially by age or sex.

**Table 1:** Population characteristics overall and by quartile of gamma gap, weighted mean (SE) or %.

		Quartiles of Gamma Gap, g/dl			
	Overall (N = 12,194*)	1.7–2.7 (N = 2,778*)	2.8–3.0 (N = 3,349*)	3.1–3.2 (N = 2,248*)	3.3–7.9 (N = 3,819*)
Age, yr	46.0 (0.3)	46.1 (0.4)	45.2 (0.3)	45.6 (0.4)	46.9 (0.4)
Women, %	51.6	43.1	50.6	57.2	59.1
Race/ethnicity, %					
Non-Hispanic White	72.8	87.9	77.8	69.4	51.2
Non-Hispanic Black	10.1	3.1	6.2	10.8	22.7
Mexican American	7.1	4.0	6.6	8.5	10.3
Hispanic	5.6	2.3	4.9	6.5	9.5
Other	4.4	2.7	4.5	4.7	6.2
Hypertension, %	35.2	31.2	32.8	36.4	42.2
Self-reported cancer, %	8.0	8.8	7.9	7.6	7.4
Body Mass Index, kg/m^2^	28.1 (0.1)	27.1 (0.2)	27.8 (0.1)	28.6 (0.2)	29.2 (0.2)
Smoking status					
Never, %	50.1	46.1	49.5	50.9	54.9
Former, %	25.4	27.3	25.3	24.6	23.8
Current, %	24.5	26.6	25.2	24.5	21.2
eGFR mL/min per 1.73m^2^	94.1 (0.4)	91.9 (0.6)	94.5 (0.5)	95.0 (0.6)	95.4 (0.6)
Albuminuria (ACR)†, mg/g	7.1	6.1	6.9	7.2	9.1
Total cholesterol, mg/dl	202.9 (0.7)	198.4 (0.8)	204.4 (1.1)	205.6 (1.2)	204.6 (1.0)
HDL cholesterol, mg/dl	52.2 (0.3)	52.7 (0.4)	52.5 (0.4)	51.5 (0.5)	51.8 (0.3)
AST, U/L	24.8 (0.2)	23.9 (0.1)	24.7 (0.4)	24.0 (0.2)	26.5 (0.4)
ALT, U/L	26.1 (0.3)	24.9 (0.3)	26.0 (0.4)	25.2 (0.3)	28.3 (1.1)
Alkaline phosphatase, U/L	71.7 (0.6)	66.8 (0.6)	70.4 (0.8)	72.7 (0.9)	78.5 (0.8)
Total bilirubin, mg/dl	0.70 (0.01)	0.75 (0.01)	0.71 (0.01)	0.67 (0.01)	0.66 (0.01)
C-reactive protein†, mg/dl	0.2	0.1	0.2	0.3	0.3
White blood cell count, SI	7.27 (0.04)	7.06 (0.04)	7.21 (0.06)	7.37 (0.07)	7.50 (0.06)
HBV core antibody positive, %	5.8	3.1	5.0	6.7	9.3
HCV antibody positive, %	1.9	0.8	1.7	1.7	3.7
HIV antibody positive‡, %	0.4‡	0.0‡	0.1‡	0.4‡	1.3‡
Total protein, g/dl	7.33 (0.01)	6.96 (0.01)	7.25 (0.01)	7.47 (0.01)	7.79 (0.01)
Serum albumin, g/dl	4.34 (0.01)	4.43 (0.01)	4.34 (0.01)	4.32 (0.01)	4.24 (0.01)

*Abbreviations*: HDL, high density lipoprotein; HBV, hepatitis B virus; HCV, hepatitis C virus; HIV, human immunodeficiency virus

*Unweighted number

^†^Median values are shown because of skewed distributions

^‡^HIV status was only available in 20–49 year-olds; unweighted number, N = 6,371.

**Fig 1:**
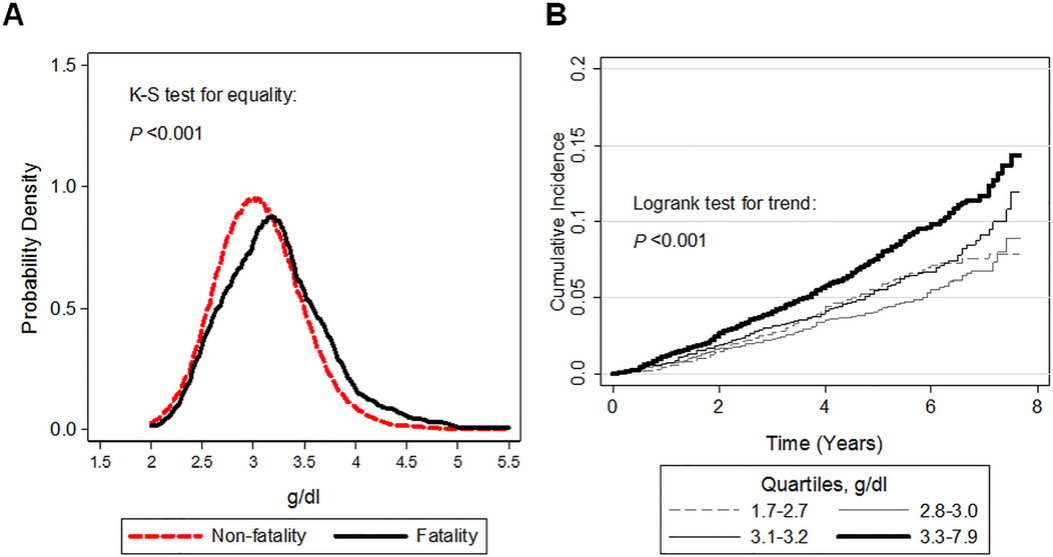
(**A**) Kernel density plots by vital status with comparison via an unweighted, two-sample, Kolmogorov–Smirnov equality-of-distributions test. (**B**) Unadjusted Kaplan-Meier cumulative incidence curve with follow-up years as the time axis and all-cause mortality as the outcome stratified by quartiles of baseline gamma gap measurements. Trend across quartiles was determined via the logrank test.

The median follow-up period was 4.8 years (IQR: 3.3 to 6.2), and there were 723 deaths (unweighted number). The 5-year unadjusted mortality rate across quartiles of gamma gap was 5.7%, 4.2%, 5.5%, and 7.8% (quartiles 1–4, respectively). There was a significant difference between median values of gamma gap by case status (*P* < 0.001) (Fig 1A). Kaplan-Meier curves revealed a significant trend across quartiles of the gamma gap (Fig 1B). Causes of death were tabulated across quartiles of gamma gap (**Table B in** S1 File), and the majority of deaths (i.e. 308 of 723) occurred in participants with a gamma gap in the highest quartile at baseline.

We evaluated different cut points that might be used to define a positive gamma gap. Gamma gap as a dichotomous variable was associated with all-cause mortality when defined at values 2.9 g/dl or greater after adjustment for age, sex, and race/ethnicity (Table 2). This corresponded to the 39^th^ percentile of adults in our population. In general, the magnitude of risk was greater with higher cut points. When adjusted for all covariates (Model 2), the range of cut points significantly associated with mortality was between 3.1 (59^th^ percentile) and 3.7 g/dl (95^th^ percentile). Notably, cut points 3.8 g/dl or greater were not associated with all-cause mortality with the exception of 4.2 g/dl, which represented the 99^th^ percentile (HR 1.74, 95% CI: 1.12, 2.73). The traditional value used to define a positive gamma gap, i.e. 4.0 g/dl, was not associated with all-cause mortality after adjustment for all covariates (HR: 1.39; 95% CI: 0.91, 2.10). However, there were fewer fatalities with higher cut points.

**Table 2:** Association between gamma gap and all-cause mortality with gamma gap dichotomized at different cutpoints (Hazard Ratios, 95% CI).

Dichotomous cut point (greater than or equal to value listed)	Corresponding percentiles	Unweighted number of deaths	Model 1 HR (95% CI)	Model 2 HR (95% CI)
2.5	8–12	691	**1.62 (1.02, 2.56)**	1.32 (0.87, 2.00)
2.6	13–19	666	1.09 (0.81, 1.46)	0.89 (0.66, 1.19)
2.7	20–28	630	1.25 (0.92, 1.71)	0.99 (0.72, 1.37)
2.8	29–38	591	1.27 (0.96, 1.67)	1.04 (0.79, 1.37)
2.9	39–48	550	**1.34 (1.07, 1.68)**	1.11 (0.89, 1.39)
3.0	49–58	496	**1.40 (1.14, 1.73)**	1.20 (0.98, 1.46)
3.1	59–67	442	**1.54 (1.27, 1.88)**	**1.30 (1.08, 1.55)**
3.2	68–75	387	**1.58 (1.27, 1.96)**	**1.33 (1.09, 1.62)**
3.3	76–82	308	**1.56 (1.28, 1.91)**	**1.29 (1.06, 1.58)**
3.4	83–87	253	**1.57 (1.26, 1.95)**	**1.26 (1.01, 1.56)**
3.5	88–91	205	**1.65 (1.30, 2.10)**	**1.29 (1.00, 1.67)**
3.6	92–94	169	**1.80 (1.36, 2.36)**	**1.38 (1.03, 1.85)**
3.7	95	130	**1.98 (1.48, 2.66)**	**1.49 (1.09, 2.04)**
3.8	96–97	102	**1.99 (1.42, 2.77)**	1.40 (0.97, 2.03)
3.9	97.5*	76	**2.11 (1.46, 3.05)**	1.47 (0.98, 2.20)
4.0	98	61	**2.08 (1.43, 3.05)**	1.39 (0.91, 2.10)
4.1	98.5*	50	**1.94 (1.21, 3.09)**	1.40 (0.84, 2.32)
4.2	99	42	**2.47 (1.62, 3.77)**	**1.74 (1.12, 2.73)**
4.3	>99	33	**2.70 (1.71, 4.28)**	1.59 (0.92, 2.75)

Note: **Bold** represents P < 0.05

Model 1: adjusted for age, sex, race/ethnicity

Model 2: adjusted for model 1 + estimated glomerular filtration rate, albuminuria, hypertension, smoking status, body mass index, total cholesterol, HDL-cholesterol, self-reported cancer, aspartate aminotransferase, alanine aminotransferase, total bilirubin, alkaline phosphatase, hepatitis B virus core Igg status, hepatitis C virus Igg status, C-reactive protein, white blood cell count, and serum albumin

*Between percentiles; 0.5 was used to indicate that this was between percentiles.

**Fig 2:**
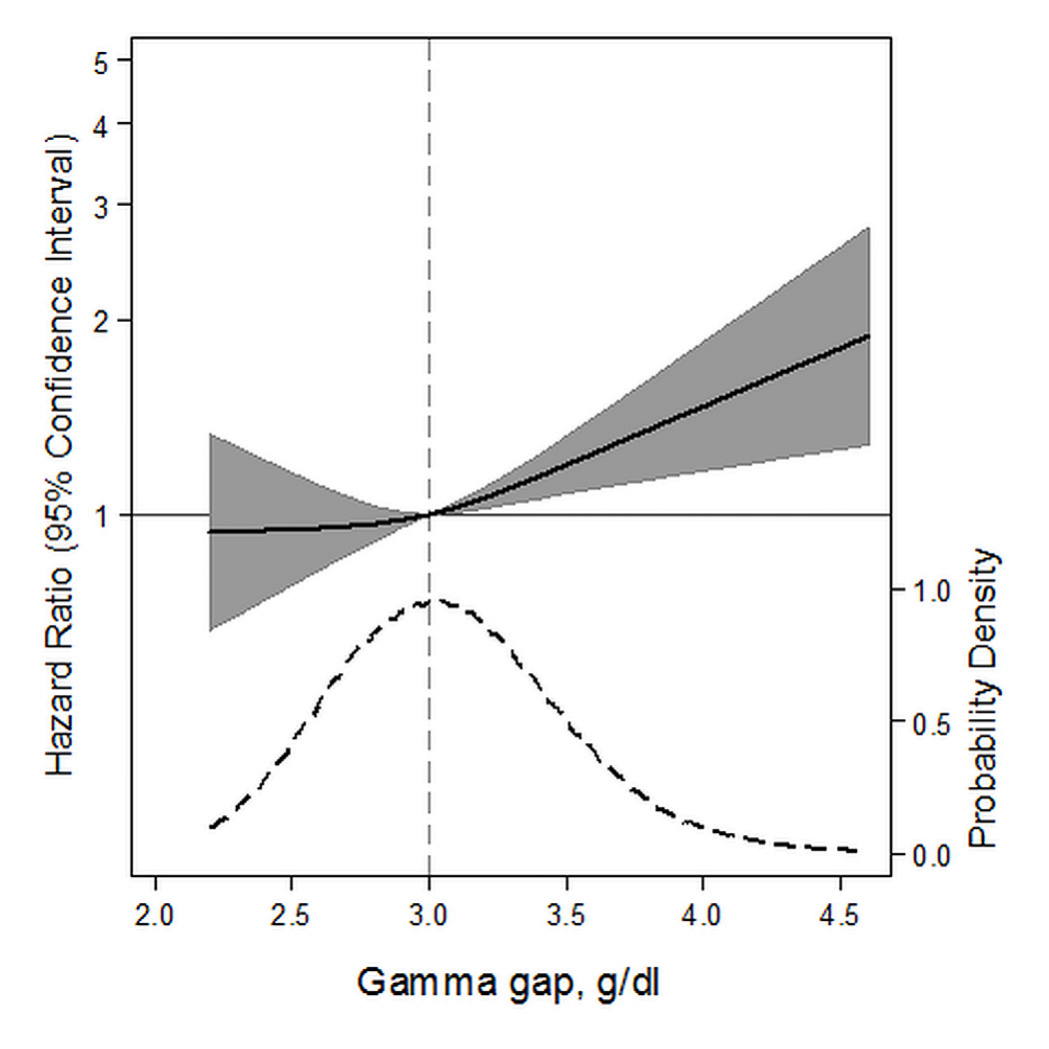
Adjusted hazard ratios (solid line) for all-cause mortality according to baseline concentrations of gamma gap from a restricted cubic spline model. Shaded region represents the 95% confidence intervals. This model was expressed relative to the 50^th^ percentile of the gamma gap with three knots placed according to Harrell’s percentiles. This model was adjusted for age, sex, race, estimated glomerular filtration rate, albuminuria, hypertension, smoking status, body mass index, total cholesterol, HDL-cholesterol, self-reported cancer, aspartate aminotransferase, alanine aminotransferase, total bilirubin, alkaline phosphatase, hepatitis B virus core Igg status, hepatitis C virus Igg status, C-reactive protein, white blood cell count, and serum albumin. The plot was truncated at the 0.5^th^ and 99.5^th^ percentiles of the gamma gap. The hazard ratios are shown on a natural log scale. This Fig is overlaid with a kernel density plot, showing the overall distribution of the baseline gamma gap. A vertical gray line represents the median value.

**Fig 3:**
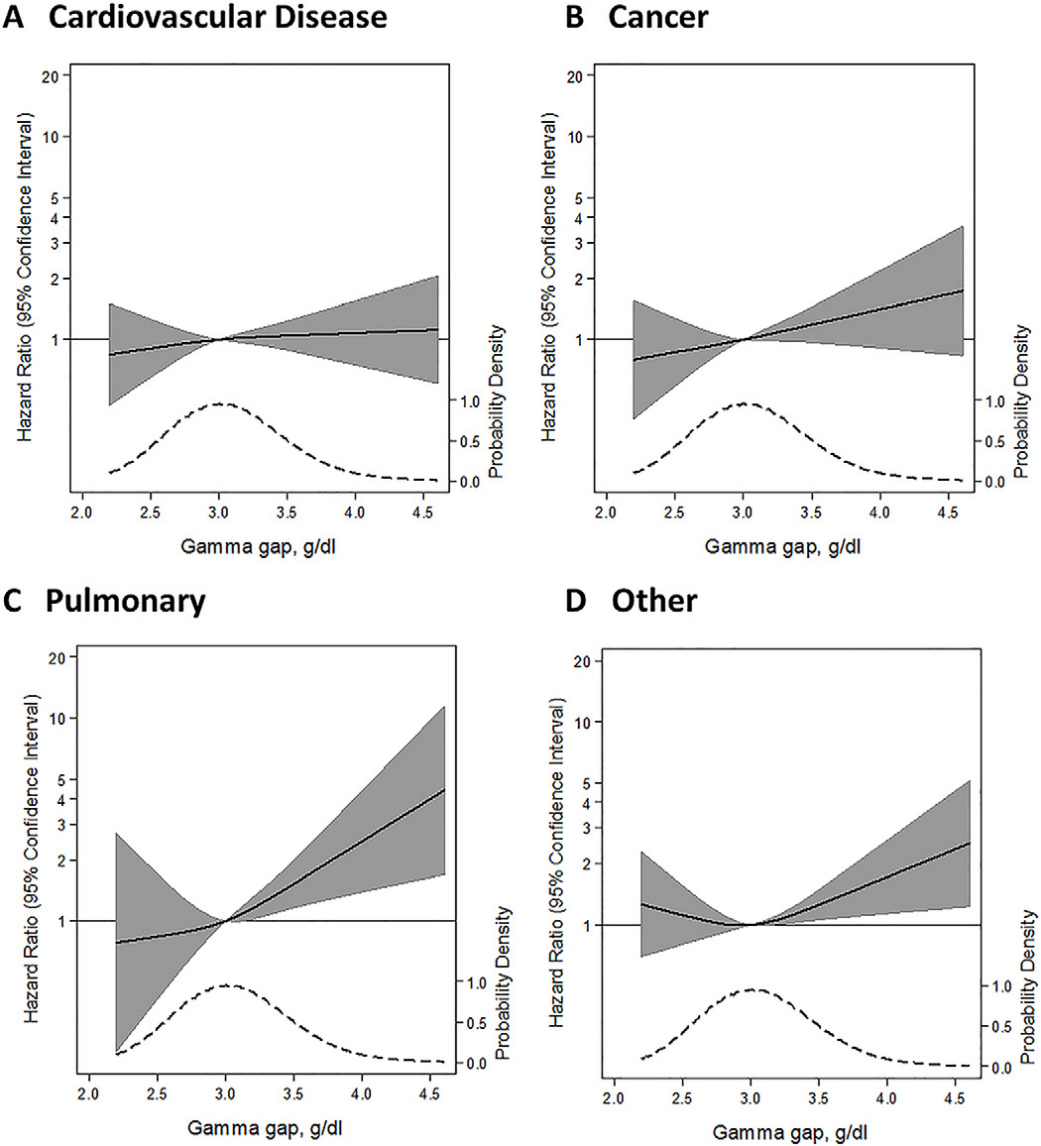
Adjusted hazard ratios (solid line) for (A) cardiovascular disease-related mortality, (B) cancer-related mortality, (C) pulmonary disease mortality, or (D) all other causes of mortality according to baseline concentrations of gamma gap from a restricted cubic spline model. Shaded region represents the 95% confidence intervals. This model was expressed relative to the 50^th^ percentile of the gamma gap with three knots placed according to Harrell’s percentiles. All four models were adjusted for age, sex, race, estimated glomerular filtration rate, albuminuria, hypertension, smoking status, body mass index, total cholesterol, HDL-cholesterol, self-reported cancer, aspartate aminotransferase, alanine aminotransferase, total bilirubin, alkaline phosphatase, hepatitis B virus core Igg status, hepatitis C virus Igg status, C-reactive protein, white blood cell count, and serum albumin. Plots were truncated at the 0.5^th^ and 99.5^th^ percentiles of the gamma gap. The hazard ratios are shown on a natural log scale. Each figure is overlaid with a kernel density plot, showing the overall distribution of the baseline gamma gap.

A higher gamma gap (per 1 g/dl) was significantly associated with a higher risk of death from any cause (HR 1.36; 95% CI: 1.10, 1.67; *P* = 0.005) (Table 3). There was also a significant trend across quartiles of gamma gap (*P* = 0.04). Similarly, a spline of the association demonstrated a nearly linearly shaped curve with risk being significantly higher above the median value (Fig 2). We examined the association between the gamma gap and specific causes of death. The gamma gap was significantly associated with death from pulmonary causes (per 1 g/dl higher gamma gap, HR 2.22; 95% CI: 1.19, 4.17; *P* = 0.01), but not cardiovascular disease or cancer. Spline models of the gamma gap showed no association with mortality from cardiovascular disease, a non-significant, positive trend for mortality from cancer, and a significant positive association with mortality from pulmonary disease (Fig 3A–3D). A sensitivity analysis comparing the higher quartile of gamma gap to the second quartile (rather than the first) did not significantly alter our findings (**Table C in** S1 File).

**Table 3:** Association between gamma gap and all-cause mortality, cardiovascular disease mortality, cancer mortality, pulmonary mortality, and other causes of mortality (Hazard Ratios, 95% CI).

	Hazard Ratio (95% CI)				
	All-cause (N = 723)	CVD (N = 258)	Cancer (N = 189)	Pulmonary (N = 80)	Other* (N = 196)
**Gamma gap, continuous variable (per 1 g/dl)**	1.36 (1.10, 1.67)	1.12 (0.81, 1.56)	1.39 (0.92, 2.08)	2.22 (1.19, 4.17)	1.41 (1.00, 1.99)
*P* value	0.005	0.48	0.11	0.01	0.05
**Gamma gap, dichotomized ≥3.0 g/dl (the median value)**	1.20 (0.98, 1.46)	1.11 (0.80, 1.54)	1.29 (0.82, 2.04)	2.14 (1.19, 3.83)	0.99 (0.70, 1.41)
*P* value	0.07	0.54	0.26	0.01	0.97
**Gamma gap, dichotomized ≥3.7 g/dl (95th percentile)**	1.49 (1.09, 2.04)	1.21 (0.76, 1.93)	1.41 (0.76, 2.59)	2.50 (0.92, 6.84)	1.67 (1.02, 2.75)
*P* value	0.01	0.41	0.26	0.072	0.042
**Gamma gap, dichotomized ≥4.0 g/dl (traditional definition)**	1.39 (0.91, 2.10)	0.85 (0.40, 1.80)	0.85 (0.41, 1.79)	1.83 (0.38, 8.79)	2.73 (1.39, 5.35)
*P* value	0.12	0.66	0.67	0.44	0.004
**Gamma gap, dichotomized ≥4.2 g/dl (99th percentile)**	1.74 (1.12, 2.73)	1.48 (0.68, 3.26)	0.90 (0.33, 2.45)	1.59 (0.27, 9.38)	3.18 (1.76, 5.75)
*P* value	0.02	0.32	0.83	0.60	<0.001
**Gamma gap, quartiles (g/dl)**					
1.7–2.7	1.0 (reference)	1.0 (reference)	1.0 (reference)	1.0 (reference)	1.0 (reference)
2.8–3.0	0.84 (0.60, 1.18)	0.75 (0.47, 1.20)	0.80 (0.43, 1.49)	1.19 (0.48, 2.92)	0.86 (0.46, 1.60)
3.1–3.2	1.09 (0.81, 1.46)	1.17 (0.68, 1.99)	1.30 (0.80, 2.12)	1.30 (0.53, 3.17)	0.73 (0.38, 1.40)
3.3–7.9	1.24 (0.92, 1.68)	0.98 (0.62, 1.54)	1.38 (0.80, 2.37)	2.23 (1.00, 5.00)	1.16 (0.70, 1.94)
*P* trend across categories as ordinal variable	0.04	0.71	0.10	0.02	0.40

All models adjusted for age, sex, race, estimated glomerular filtration rate, albuminuria, hypertension, smoking status, body mass index, total cholesterol, HDL-cholesterol, self-reported cancer, aspartate aminotransferase, alanine aminotransferase, total bilirubin, alkaline phosphatase, hepatitis B virus core Igg status, hepatitis C virus Igg status, C-reactive protein, white blood cell count, and serum albumin

*Causes of death other than cardiovascular disease, cancer, or pulmonary disease

*Abbreviations*: N represents the unweighted number of deaths; CVD represents cardiovascular disease.

Sensitivity analyses were performed restricting the study population to participants with a negative HIV screening test (unweighted N = 6,334) (**Table D in** S1 File). There were a total of 57 deaths in this subgroup. Despite this large loss of power, there was a non-significant, positive association between gamma gap and all-cause mortality (HR: 1.76; *P* = 0.17). Restricting the population to participants without hepatitis B virus or hepatitis C virus antibodies did not attenuate our findings. In a subgroup restricted to participants without HIV and without hepatitis B virus or hepatitis C virus antibodies (total deaths were 50), gamma gap also demonstrated a non-significant, positive association with death from any cause (HR: 1.41; *P* = 0.52).

## Discussion

This represents the first formal evaluation of the association between gamma gap and a clinical outcome in a general US population, despite its frequent use in clinical settings. Gamma gap was strongly associated with death from any cause over a short time period in a non-institutionalized, general population even after adjustment for multiple risk factors. As opposed to the traditional clinical practice of defining a gamma gap at a value of 4.0 g/dl, gamma gap was associated with increased risk of mortality at values as low as 3.1 g/dl. Unexpectedly, death from pulmonary causes was most strongly associated with gamma gap.

Despite widespread application of the gamma gap in clinical practice there is currently very little literature guiding its use. Increased gamma gap has been shown to be strongly associated with a positive serum or urine protein electrophoresis testing [1]. Further, prospective studies have shown a relationship between total protein and mortality in transplant patients [11], total protein with low albumin and mortality in hospitalized patients [12], albumin-to-globulin ratio and mortality in cancer patients [13–15], IgA levels and cancer-related mortality in elderly patients [16], and hypergammaglobulinemia and decreased survival in patients with rheumatologic conditions [17,18]. A study of patients admitted to a burn unit with serial measures of serum albumin and protein, showed that developing a lower albumin-to-globulin ratio, which would be equivalent to a greater gamma gap, was associated with higher mortality [19]. Moreover, a recent actuarial manuscript, using billing data described serum globulins as an important predictor of mortality [20]. Our manuscript represents the first prospective study to look specifically at the gamma gap and its relationship with all-cause and specific causes of mortality in a general population.

We can speculate as to the mechanism by which the gamma gap is associated with mortality. One possibility is that it reflects inflammation, which is associated with an increase in serum acute-phase reactant proteins such as c-reactive protein [21] and a decrease in albumin [22]. Another mechanism may be due to secondary conditions like amyloidosis [18] that result from the increased production of immunoglobulins, seen in hematopoietic neoplasms, infections, or rheumatologic conditions [23].

A positive gamma gap is traditionally defined at a value of 4.0 g/dl or greater, which corresponded to the 98^th^ percentile in the US adult population. Our data shows that in fully adjusted models, lower cut points, ranging from 3.1 to 3.7 g/dl, are associated with an increased risk of death, but not the traditional value of 4.0 g/dl. It is apparent that there were fewer fatalities at higher cut points, which may result in over-fitting of models at higher levels of the gamma gap. This may explain why cut points above 3.7 g/dl were non-significant in the fully adjusted model. However, given the linear relationship between gamma gap and risk of mortality, it is also evident that having a cut point of 4.0 g/dl may be too high, missing persons at risk of death in the 3.1 to 3.9 g/dl range. It should also be noted that some studies utilize a percentile-based approach to defining elevated biomarkers, such as the 99^th^ percentile [24], which would correspond to a value of 4.2 g/dl. Notably, gamma gap defined at 4.2 g/dl was also strongly associated with mortality even in the fully adjusted model.

Gamma gap was strongly associated with mortality from pulmonary disease. Upon closer inspection of the deaths from pulmonary disease, this association was driven in part by death from pneumonia. Interestingly, among cancer deaths, death from neoplasms of the trachea, bronchus, or lung also demonstrated a crude, increasing trend across quartiles of gamma gap. The biologic causes of these associations are unknown. A number of prior studies have described low albumin being associated with perioperative pulmonary complications [25–30], however, these studies did not examine the gamma gap, which was associated with mortality from a pulmonary cause in our study independent of albumin. One small study of children with cystic fibrosis found that lower levels of serum immunoglobulins were associated with a lower risk of pneumonia [31]. Further, other disease states characterized by excess immunoglobulins are associated with pneumonia such as multiple myeloma [32]. Whether an elevated gamma gap is a cause of death from pulmonary causes, versus merely a marker of risk, is beyond the scope of this study, but is an important question for future research.

We did not find differences in the population distribution of gamma gap by age or sex, suggesting that the synthesis and maintenance of serum total protein or albumin levels are not affected by age or sex in a general population. However, as we did not have repeat measures of total protein and albumin, we cannot confirm that serum levels do not decline with age. Further research employing repeated, within person measures, are needed to examine the change in gamma gap over one’s lifetime.

This study has a number of limitations. First, gamma gap was based on a single measurement, so we could not assess changes over time or within-person variability. Second, human immunodeficiency virus was only measured on 20–49 year-olds. While there was a low prevalence of human immunodeficiency infections in US adults over age 50 years in 1999–2004, we could not adjust for HIV exposure in our models. Further, few adults age 20–49 proceeded to die during our follow-up period, reducing our statistical power in the subgroup analysis of adults without human immunodeficiency exposure. Despite this, we still observed a non-significant positive association between gamma gap and mortality. Third, while the association between gamma gap and death from any cause demonstrates its utility for risk stratification, it does not clarify the biologic pathways behind the relationship. Furthermore, cause of death based on ICD10 codes from death certificates is prone to misclassification (a common problem with medical records due to lack of an adjudication mechanism) likely attenuating our results. Fourth, there were a small number of deaths in our relatively healthy, non-institutionalized study population, which limited our ability to examine deaths from other common causes such as diabetes, kidney disease, or liver disease. Finally, residual confounding is always a concern with observational studies.

This study has a number of strengths. We used a large, well-established, highly generalizable study population, representing the demographic constituents of the US. Data assessments were comprehensive, including questionnaires, physical exams, and laboratory measures. Furthermore, the study was executed with standardized, high quality measures and evaluated a clinically important outcome, mortality.

In conclusion, gamma gap is strongly associated with death from any cause and more specifically, death from pulmonary disease over a short time period. Gamma gap is useful for risk stratification at values lower than the traditional definition. Further studies should examine the biologic pathways for this association as well as examine the effects of changes in gamma gap over time.

## Supporting Information

S1 File **Table A**. Weighted percentiles of gamma gap (g/dl) in the US adult population by age and sex. **Table B**. Number of each cause of death (with corresponding ICD10 code) overall and by quartile of gamma gap. **Table C**. Association between gamma gap and all-cause mortality, cardiovascular disease mortality, cancer mortality, pulmonary mortality, and other causes of mortality (Hazard Ratios, 95% CI). **Table D**. Association between gamma gap and all-cause mortality (Hazard Ratios, 95% CI), restricted to participants with a negative HIV screening test, negative hepatitis B virus core antibody, or negative hepatitis C virus antibody.
(DOCX)
