# Supplementary material for: The Gamma Gap and All-Cause Mortality
Source: PLoS One. 2015 Dec 2;10(12):e0143494. doi: 10.1371/journal.pone.0143494 (PMC4668045; doi:10.1371/journal.pone.0143494)
Supplement: S1 File — Table A. Weighted percentiles of gamma gap (g/dl) in the US adult population by age and sex. Table B. Number of each cause of death (with corresponding ICD10 code) overall and by quartile of gamma gap. Table C. Association between gamma gap and all-cause mortality, cardiovascular disease mortality, cancer mortality, pulmonary mortality, and other causes of mortality (Hazard Ratios, 95% CI). Table D. Association between gamma gap and all-cause mortality (Hazard Ratios, 95% CI), restricted to participants with a negative HIV screening test, negative hepatitis B virus core antibody, or negative hepatitis C virus antibody. (DOCX) [file pone.0143494.s001.docx]

**Supporting Information S1**

**Tables A-D**

| **S1 Table A. Weighted percentiles of gamma gap (g/dl) in the US adult population by age and sex** | | | | | | | | |
| --- | --- | --- | --- | --- | --- | --- | --- | --- |
|  |  | **1st** | **5th** | **25th** | **50th** | **75th** | **95th** | **99th** |
| Overall |  | 2.1 | 2.4 | 2.7 | 3.0 | 3.2 | 3.7 | 4.2 |
|  | Male | 2.1 | 2.3 | 2.7 | 2.9 | 3.2 | 3.6 | 4.1 |
|  | Female | 2.2 | 2.4 | 2.8 | 3.0 | 3.3 | 3.8 | 4.2 |
| Male |  |  |  |  |  |  |  |  |
|  | 20-29 yr | 2.1 | 2.4 | 2.7 | 2.9 | 3.2 | 3.6 | 3.9 |
|  | 30-39 yr | 2.1 | 2.3 | 2.7 | 2.9 | 3.2 | 3.5 | 3.9 |
|  | 40-49 yr | 2.2 | 2.3 | 2.7 | 2.9 | 3.2 | 3.6 | 4.1 |
|  | 50-59 yr | 2.0 | 2.3 | 2.6 | 2.9 | 3.2 | 3.6 | 3.9 |
|  | 60-69 yr | 2.1 | 2.4 | 2.7 | 3.0 | 3.2 | 3.8 | 4.7 |
|  | 70-79 yr | 2.1 | 2.3 | 2.7 | 3.0 | 3.3 | 3.7 | 4.2 |
|  | ≥80 yr | 2.2 | 2.4 | 2.7 | 3.0 | 3.3 | 3.8 | 4.3 |
| Female |  |  |  |  |  |  |  |  |
|  | 20-29 yr | 2.2 | 2.5 | 2.8 | 3.0 | 3.3 | 3.7 | 4.1 |
|  | 30-39 yr | 2.2 | 2.5 | 2.8 | 3.0 | 3.3 | 3.7 | 4.1 |
|  | 40-49 yr | 2.2 | 2.4 | 2.8 | 3.0 | 3.3 | 3.7 | 4.2 |
|  | 50-59 yr | 2.1 | 2.4 | 2.7 | 3.0 | 3.3 | 3.9 | 4.5 |
|  | 60-69 yr | 2.1 | 2.3 | 2.7 | 3.0 | 3.3 | 3.8 | 4.2 |
|  | 70-79 yr | 2.2 | 2.4 | 2.7 | 3.0 | 3.3 | 3.9 | 4.5 |
|  | ≥80 yr | 2.1 | 2.4 | 2.7 | 3.0 | 3.3 | 3.8 | 4.3 |

| **S1 Table B. Number of each cause of death (with corresponding ICD10 code) overall and by quartile of gamma gap** | | | | | | | | |
| --- | --- | --- | --- | --- | --- | --- | --- | --- |
|  | |  |  |  | **Number of Deaths by Quartiles of Gamma Gap, g/dl** | | | |
|  | |  | **Overall** |  | **1.7 - 2.7** | **2.8 - 3.0** | **3.1 - 3.2** | **3.3 - 7.9** |
| **Death from any cause** | | | **723** |  | **132** | **149** | **134** | **308** |
|  | | |  |  |  |  |  |  |
| **Cardiovascular disease** | | | **258** |  | **54** | **53** | **47** | **104** |
|  | Acute rheumatic fever and chronic rheumatic heart diseases (I00-I09) | | 2 |  | 0 | 1 | 0 | 1 |
|  | Hypertensive heart disease (I11) | | 6 |  | 1 | 0 | 1 | 4 |
|  | Hypertensive heart and renal disease (I13) | | 1 |  | 0 | 1 | 0 | 0 |
|  | Acute myocardial infarction (I21-I22) | | 44 |  | 6 | 12 | 11 | 15 |
|  | Atherosclerotic cardiovascular disease, so described (I25.0) | | 17 |  | 4 | 4 | 0 | 9 |
|  | All other forms of chronic ischemic heart disease (I20,I25.1-I25.9) | | 64 |  | 16 | 11 | 14 | 23 |
|  | Heart failure (I50) | | 25 |  | 4 | 4 | 4 | 13 |
|  | All other forms of heart disease (I26-I28,I34-I38,I42-I49,I51) | | 31 |  | 6 | 2 | 4 | 19 |
|  | Essential (primary) hypertension and hypertensive renal disease (I10,I12) | | 7 |  | 2 | 3 | 1 | 1 |
|  | Cerebrovascular diseases (I60-I69) | | 49 |  | 11 | 13 | 10 | 15 |
|  | Atherosclerosis (I70) | | 7 |  | 3 | 2 | 2 | 0 |
|  | Aortic aneurysm and dissection (I71) | | 2 |  | 0 | 0 | 0 | 2 |
|  | Other diseases of arteries, arterioles and capillaries (I72-I78) | | 1 |  | 0 | 0 | 0 | 1 |
|  | Other disorders of circulatory system (I80-I99) | | 2 |  | 1 | 0 | 0 | 1 |
|  |  | |  |  |  |  |  |  |
| **Cancer** | | | **189** |  | **34** | **36** | **41** | **78** |
|  | Malignant neoplasm of esophagus (C15) | | 1 |  | 0 | 0 | 1 | 0 |
|  | Malignant neoplasm of stomach (C16) | | 4 |  | 0 | 1 | 2 | 1 |
|  | Malignant neoplasms of colon, rectum and anus (C18-C21) | | 19 |  | 3 | 2 | 7 | 7 |
|  | Malignant neoplasms of liver and intrahepatic bile ducts (C22) | | 4 |  | 2 | 0 | 0 | 2 |
|  | Malignant neoplasm of pancreas (C25) | | 8 |  | 1 | 0 | 1 | 6 |
|  | Malignant neoplasm of larynx (C32) | | 1 |  | 0 | 1 | 0 | 0 |
|  | Malignant neoplasms of trachea, bronchus and lung (C33-C34) | | 68 |  | 10 | 13 | 16 | 29 |
|  | Malignant neoplasm of breast (C50) | | 8 |  | 2 | 1 | 1 | 4 |
|  | Malignant neoplasm of cervix uteri (C53) | | 1 |  | 0 | 0 | 0 | 1 |
|  | Malignant neoplasms of corpus uteri and uterus, part unspecified (C54-C55) | | 2 |  | 0 | 2 | 0 | 0 |
|  | Malignant neoplasm of ovary (C56) | | 5 |  | 1 | 1 | 1 | 2 |
|  | Malignant neoplasm of prostate (C61) | | 20 |  | 6 | 6 | 3 | 5 |
|  | Malignant neoplasms of kidney and renal pelvis (C64-C65) | | 6 |  | 0 | 2 | 0 | 4 |
|  | Malignant neoplasm of bladder (C67) | | 4 |  | 2 | 1 | 0 | 1 |
|  | Malignant neoplasms of meninges, brain and other parts of central nervous system (C70-C72) | | 2 |  | 0 | 0 | 0 | 2 |
|  | Non-Hodgkin's lymphoma (C82-C85) | | 6 |  | 1 | 1 | 3 | 1 |
|  | Leukemia (C91-C95) | | 3 |  | 2 | 0 | 0 | 1 |
|  | Multiple myeloma and immunoproliferative neoplasms (C88,C90) | | 4 |  | 0 | 0 | 1 | 3 |
|  | All other and unspecified malignant neoplasms (C17,C23-C24,C26-C31,C37-C41, C44-C49,C51-C52,C57-C60,C62-C63,C66,C68-C69,C73-C80,C97) | | 20 |  | 2 | 5 | 4 | 9 |
|  | In situ neoplasms, benign neoplasms and neoplasms of uncertain or unknown behavior (D00-D48) | | 3 |  | 2 | 0 | 1 | 0 |
|  |  | |  |  |  |  |  |  |
| **Pulmonary disease** | | | **80** |  | **16** | **16** | **14** | **34** |
|  | Pneumonia (J12-J18) | | 22 |  | 3 | 2 | 5 | 12 |
|  | Emphysema (J43) | | 4 |  | 0 | 0 | 1 | 3 |
|  | Asthma (J45-J46) | | 4 |  | 3 | 1 | 0 | 0 |
|  | Other chronic lower respiratory diseases (J44,J47) | | 36 |  | 8 | 10 | 5 | 13 |
|  | Pneumonitis due to solids and liquids (J69) | | 3 |  | 0 | 1 | 1 | 1 |
|  | Other diseases of respiratory system (J00-J06,J30-J39,J67,J70-J98) | | 11 |  | 2 | 2 | 2 | 5 |
|  |  | |  |  |  |  |  |  |
| **Other causes of death** | | | **196** |  | **28** | **44** | **32** | **92** |
|  | Certain other intestinal infections (A04,A07-A09) | | 1 |  | 0 | 0 | 0 | 1 |
|  | Septicemia (A40-A41) | | 11 |  | 1 | 3 | 2 | 5 |
|  | Viral hepatitis (B15-B19) | | 2 |  | 0 | 0 | 0 | 2 |
|  | Human immunodeficiency virus (HIV) disease (B20-B24) | | 7 |  | 0 | 0 | 1 | 6 |
|  | Other and unspecified infectious and parasitic diseases and their sequelae (A00,A05,A20-A36,A42-A44,A48-A49, A54-79,A81-A82,A85.0-A85.1,A85.8, A86-B04,B06-B09,B25-B49,B55-99) | | 4 |  | 0 | 0 | 0 | 4 |
|  | Diabetes mellitus (E10-E14) | | 24 |  | 3 | 5 | 5 | 11 |
|  | Malnutrition (E40-E46) | | 1 |  | 0 | 1 | 0 | 0 |
|  | Parkinson's disease (G20-G21) | | 5 |  | 1 | 2 | 0 | 2 |
|  | Alzheimer's disease (G30) | | 19 |  | 1 | 7 | 5 | 6 |
|  | Alcoholic liver disease (K70) | | 4 |  | 0 | 0 | 0 | 4 |
|  | Other chronic liver disease and cirrhosis (K73-K74) | | 7 |  | 0 | 1 | 0 | 6 |
|  | Cholelithiasis and other disorders of gallbladder (K80-K82) | | 1 |  | 0 | 0 | 1 | 0 |
|  | Acute and rapidly progressive nephritic and nephrotic syndrome (N00-N01,N04) | | 1 |  | 1 | 0 | 0 | 0 |
|  | Renal failure (N17-N19) | | 13 |  | 1 | 1 | 3 | 8 |
|  | Symptoms, signs and abnormal clinical and laboratory findings, not elsewhere classified (R00-R99) | | 2 |  | 0 | 1 | 1 | 0 |
|  | All other diseases (Residual) (D65-E07,E15-E34,E65-F99,G04-G12,G23-G25,G31-H93, K00-K22,K29-K31,K50-K66,K71-K72,K75-K76,K83-M99, N13.0-N13.5,N13.7-N13.9,N14,N15.0,N15.8-N15.9,N20-N23,N28-N39,N41-N64,N80-N98) | | 48 |  | 10 | 14 | 7 | 17 |
|  | Motor vehicle accidents (V02-V04,V09.0,V09.2,V12-V14,V19.0-V19.2, V19.4-V19.6,V20-V79,V80.3-V80.5,V81.0-V81.1,V82.0-V82.1,V83-V86, V87.0-V87.8,V88.0 V88.8,V89.0,V89.2) | | 10 |  | 4 | 0 | 2 | 4 |
|  | Other land transport accidents (V01,V05-V06,V09.1,V09.3-V09.9, V10-V11, V15-V18,V19.3,V19.8-V19.9,V80.0-V80.2,V80.6-V80.9,V81.2-V81.9, V82.2-V82.9,V87.9,V88.9,V89.1,V89.3,V89.9) | | 1 |  | 0 | 1 | 0 | 0 |
|  | Water, air and space, and other and unspecified transport accidents and their sequelae (V90-V99,Y85) | | 1 |  | 0 | 0 | 0 | 1 |
|  | Falls (W00-W19) | | 4 |  | 1 | 1 | 0 | 2 |
|  | Accidental poisoning and exposure to noxious substances (X40-X49) | | 3 |  | 0 | 2 | 0 | 1 |
|  | Other and unspecified nontransport accidents and their sequelae (W20-W31,W35-W64,W75-W99,X10-X39,X50-X59,Y86) | | 6 |  | 1 | 2 | 0 | 3 |
|  | Intentional self-harm (suicide) by discharge of firearms (X72-X74) | | 1 |  | 0 | 0 | 0 | 1 |
|  | Intentional self-harm (suicide) by other and unspecified means and their sequelae (*U03,X60-X71,X75-X84,Y87.0) | | 5 |  | 3 | 1 | 1 | 0 |
|  | Assault (homicide) by other and unspecified means and their sequelae (*U01.0-*U01.3,*U01.5-*U01.9,*U02,X85-X92,X96-Y09,Y87.1) | | 1 |  | 0 | 0 | 1 | 0 |
|  | Legal intervention (Y35,Y89.0) | | 1 |  | 0 | 0 | 0 | 1 |
|  | Other and unspecified events of undetermined intent and their sequelae (Y10-Y21,Y25-Y34,Y87.2,Y89.9) | | 2 |  | 1 | 1 | 0 | 0 |
|  | No classification code | | 11 |  | 0 | 1 | 3 | 7 |

| **S1 Table C. Association between gamma gap and all-cause mortality, cardiovascular disease mortality, cancer mortality, pulmonary mortality, and other causes of mortality (Hazard Ratios, 95% CI)** | | | | | | |
| --- | --- | --- | --- | --- | --- | --- |
|  |  | **Hazard Ratio (95% CI)** | | | | |
|  |  | **All-cause (N = 723)** | **CVD (N = 258)** | **Cancer (N = 189)** | **Pulmonary (N = 80)** | **Other* (N = 196)** |
| **Gamma gap, quartiles (g/dl)** | |  |  |  |  |  |
|  | 1.7 - 2.7 | 1.19 (0.85, 1.67) | 1.33 (0.83, 2.11) | 1.25 (0.67, 2.34) | 0.84 (0.34, 2.06) | 1.16 (0.63, 2.17) |
|  | 2.8 - 3.0 | 1.0 (reference) | 1.0 (reference) | 1.0 (reference) | 1.0 (reference) | 1.0 (reference) |
|  | 3.1 - 3.2 | 1.29 (0.97, 1.72) | 1.55 (0.87, 2.74) | 1.63 (0.86, 3.07) | 1.09 (0.51, 2.34) | 0.85 (0.48, 1.49) |
|  | 3.3 - 7.9 | 1.47 (1.16, 1.87) | 1.30 (0.92, 1.84) | 1.73 (0.99, 3.01) | 1.88 (1.00, 3.51) | 1.36 (0.85, 2.17) |
|  |  |  |  |  |  |  |
| All models adjusted for age, sex, race, estimated glomerular filtration rate, albuminuria, hypertension, smoking status, body mass index, total cholesterol, HDL-cholesterol, self-reported cancer, aspartate aminotransferase, alanine aminotransferase, total bilirubin, alkaline phosphatase, hepatitis B virus core Igg status, hepatitis C virus Igg status, C-reactive protein, white blood cell count, and serum albumin | | | | | | |
| *Causes of death other than cardiovascular disease, cancer, or pulmonary disease | | | | | |  |
| *Abbreviations*: N represents the unweighted number of deaths; CVD represents cardiovascular disease | | | | | | |

| **S1 Table D. Association between gamma gap and all-cause mortality (Hazard Ratios, 95% CI), restricted to participants with a negative HIV screening test, negative hepatitis B virus core antibody, or negative hepatitis C virus antibody** | | | |
| --- | --- | --- | --- |
|  |  | **Gamma gap per 1 g/dl, Hazard Ratio (95% CI)** | ***P*** |
| **HIV negative (N = 57)** | | 1.76 (0.77, 4.01) | 0.17 |
| **HBV & HCV negative (N = 624)** | | 1.40 (1.12, 1.74) | 0.004 |
| **HIV, HBV, & HCV negative (N = 50)** | | 1.41 (0.49, 4.00) | 0.52 |
|  |  |  |  |
| All models adjusted for age, sex, race, estimated glomerular filtration rate, albuminuria, hypertension, smoking status, body mass index, total cholesterol, HDL-cholesterol, self-reported cancer, aspartate aminotransferase, alanine aminotransferase, total bilirubin, alkaline phosphatase, hepatitis B virus core Igg status, hepatitis C virus Igg status, C-reactive protein, white blood cell count, and serum albumin | | | |
| *Abbreviations*: N represents the unweighted number of deaths; HIV represents human immunodeficiency virus; HBV represents hepatitis B virus; HCV represents hepatitis C virus | | | |
